# Sequential bortezomib and temozolomide treatment promotes immunological responses in glioblastoma patients with positive clinical outcomes: A phase 1B study

**DOI:** 10.1002/iid3.315

**Published:** 2020-06-24

**Authors:** Mohummad A. Rahman, Jorunn Brekke, Victoria Arnesen, Marianne H. Hannisdal, Andrea G. Navarro, Andreas Waha, Lars Herfindal, Cecilie B. Rygh, Eirik Bratland, Petter Brandal, Judit Haasz, Leif Oltedal, Hrvoje Miletic, Arvid Lundervold, Stein A. Lie, Dorota Goplen, Martha Chekenya

**Affiliations:** ^1^ Department of Biomedicine University of Bergen Bergen Norway; ^2^ Department of Oncology Haukeland University Hospital Bergen Norway; ^3^ Department of Neuropathology University of Bonn Bonn Germany; ^4^ Department of Clinical Sciences University of Bergen Bergen Norway; ^5^ Department of Radiology, Mohn Medical Imaging and Visualization Centre Haukeland University Hospital Bergen Norway; ^6^ Department of Medical Genetics Haukeland University Hospital Bergen Norway; ^7^ Department of Oncology Oslo University Hospital Oslo Norway; ^8^ Department of Pathology Haukeland University Hospital Bergen Norway; ^9^ Department of Clinical Dentistry University of Bergen Norway

**Keywords:** bortezomib, immune checkpoint, MGMT, recurrent GBM, temozolomide, Th1/Th2 cytokine ratios

## Abstract

**Background:**

Glioblastoma (GBM) is an aggressive malignant brain tumor where median survival is approximately 15 months after best available multimodal treatment. Recurrence is inevitable, largely due to O^6^ methylguanine DNA methyltransferase (*MGMT*) that renders the tumors resistant to temozolomide (TMZ). We hypothesized that pretreatment with bortezomib (BTZ) 48 hours prior to TMZ to deplete MGMT levels would be safe and tolerated by patients with recurrent GBM harboring unmethylated *MGMT* promoter. The secondary objective was to investigate whether 26S proteasome blockade may enhance differentiation of cytotoxic immune subsets to impact treatment responses measured by radiological criteria and clinical outcomes.

**Methods:**

Ten patients received intravenous BTZ 1.3 mg/m^2^ on days 1, 4, and 7 during each 4th weekly TMZ‐chemotherapy starting on day 3 and escalated from 150 mg/m^2^ per oral 5 days/wk via 175 to 200 mg/m^2^ in cycles 1, 2, and 3, respectively. Adverse events and quality of life were evaluated by CTCAE and EQ‐5D‐5L questionnaire, and immunological biomarkers evaluated by flow cytometry and Luminex enzyme‐linked immunosorbent assay.

**Results:**

Sequential BTZ + TMZ therapy was safe and well tolerated. Pain and performance of daily activities had greatest impact on patients' self‐reported quality of life and were inversely correlated with Karnofsky performance status. Patients segregated a priori into three groups, where group 1 displayed stable clinical symptoms and/or slower magnetic resonance imaging radiological progression, expanded CD4^+^ effector T‐cells that attenuated cytotoxic T‐lymphocyte associated protein‐4 and PD‐1 expression and secreted interferon γ and tumor necrosis factor α in situ and ex vivo upon stimulation with PMA/ionomycin. In contrast, rapidly progressing group 2 patients exhibited tolerised T‐cell phenotypes characterized by fourfold to sixfold higher interleukin 4 (IL‐4) and IL‐10 Th‐2 cytokines after BTZ + TMZ treatment, where group 3 patients exhibited intermediate clinical/radiological responses.

**Conclusion:**

Sequential BTZ + TMZ treatment is safe and promotes Th1‐driven immunological responses in selected patients with improved clinical outcomes (Clinicaltrial.gov (NCT03643549)).

## Graphical abstract

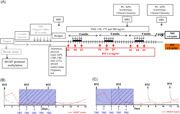

## 1 INTRODUCTION

Glioblastoma (GBM) is the most prevalent and aggressive brain tumor in humans. Standard first‐line treatment for fit patients consists of surgery aiming for maximal safe tumor resection,^1^, ^2^ followed by radiochemotherapy with daily temozolomide (TMZ) administered concomitantly with external beam fractionated ionizing radiation for 6 weeks to a total dose of 60 Gy in 30 fractions. Adjuvant TMZ is administered for 5 days every 4th week for up to six cycles.^3^ Despite this multimodal treatment, median overall survival is only approximately 15 months.^3^, ^4^, ^5^ For patients harboring GBM with unmethylated O^6^ methylguanine DNA methyltransferase (*MGMT*) promoter, 2‐year survival rate is only 14% compared to 46% for those with methylated *MGMT* promoter^6^ and thus, *MGMT* promoter status is both prognostic and predictive of treatment response to TMZ chemotherapy. Tumor recurrence is inevitable and median time to neoplastic progression is approximately 6.9 months.^3^ There is no standard treatment after recurrence, therefore, options are dependent on physician's choice or practice at the given institution. The European society for medical oncology guidelines recommend that patients be treated within investigational protocols.^7^

Profound cellular and molecular heterogeneity typifies GBM^8^, ^9^ thus, combination therapy to mitigate drug resistance has become the benchmark of neuro‐oncology. Nevertheless, the alkylating chemotherapeutic agents, temozolomide and lomustine are still the backbone of systemic glioma therapy.^10^ Several clinical studies have explored blocking of MGMT to improve TMZ efficacy in treatment resistant GBM.^11^, ^12^, ^13^, ^14^ Transcription factors and coactivators, including nuclear factor κB (NF‐κB),^15^ tumor protein 53 (TP53),^16^, ^17^ transcription factor Sp1,^16^ hypoxia inducible factor‐1α (HIF‐1α),^18^ cyclic adenosine monophosphate response element‐binding protein,^19^ MGMT enhancer binding protein^20^ and signal transducer and activator of transcription 3 (STAT3)^21^ have also been investigated for their potential to regulate MGMT protein expression. It has been established that NF‐κB binding sites are present within the *MGMT* promoter region, that *MGMT* messenger RNA (mRNA) is induced by NF‐κB/p65, and that MGMT expression correlates with NF‐κB activation regardless of promoter methylation status.^15^, ^22^ NF‐κB activation requires 26S proteasomal processing.^23^

Bortezomib (BTZ) is a proteasome inhibitor that has been approved for treatment of multiple myeloma, mantle cell lymphoma and trialed in early phase trials for treatment of GBM. It blocks the chymotryptic activity of the 26S proteasome and prevents degradation of misfolded proteins or abundant short‐lived proteins such as transcription factors that may be important in regulation of tumor and immune cell differentiation, proliferation, and apoptosis. We recently showed that GBM pretreatment with BTZ for 48 hours prohibited phosphorylation of IkBα, resulting in reduced nuclear translocation of the activated phosphorylated NF‐κB p65/RelA subunit. This correlated with reduced MGMT protein and mRNA expression by 70%‐80% and sensitized the GBM cells to TMZ chemotherapy.^22^ Two other studies also investigated MGMT depletion via mechanisms associated with activation of NFκB, MAPK, STAT3, and HIF‐1α pathways after BTZ treatment.^16^, ^18^ As well as depleting the tumor's cytoprotective mechanisms, agents that simultaneously promote tumor recognition by cells of the immune system may be attractive anticancer candidates. Indeed, we demonstrated that natural killer (NK) cells treated with BTZ exhibited more mature, activated and cytotoxic CD57^+^CD16^dim^CD69^+^ phenotype, and that efficacy of combination treatment of GBM cells with BTZ + NK cells in vitro and in vivo was augmented by tumor necrosis factor related ligand (TRAIL)‐receptor interactions, as well as tumor expression of stress‐ligands recognized by activating NKG2D receptor.^24^ Sequential combination of BTZ 48 hours before TMZ 164 mg/m^2^ treatment, depleted MGMT mRNA in vivo, attenuated tumor growth and significantly prolonged animal survival.^22^ Thus, this phase IB of our BORTEM‐17 clinical trial (NCT03643549) was launched to investigate whether pretreatment of recurrent GBM patients with BTZ 1.3 mg/m^2^ on days 1, 4, and 7, to deplete MGMT levels 48 hours before TMZ, commencing on days 3 to 7 every 4th week, might sensitize *MGMT* unmethylated GBM to TMZ chemotherapy. We assessed the maximal safe escalated TMZ dose administered in sensitization schedule with BTZ and whether treatment enhanced differentiation of cytotoxic immune subsets to impact treatment responses.

## 2 MATERIALS AND METHODS

### 2.1 Treatment plan

**Figure 1:**
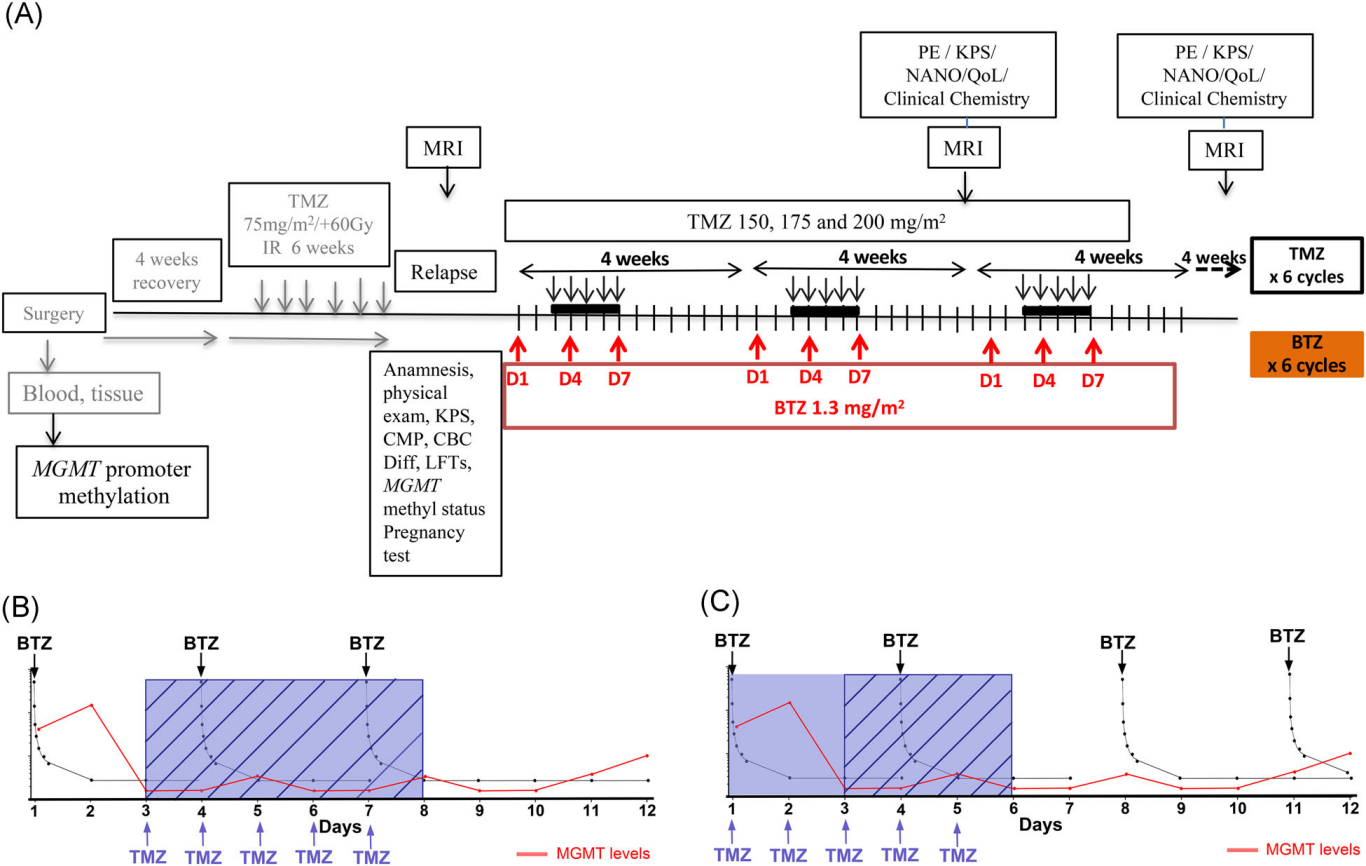
Schematic of trial schedule. A, Timeline showing BORTEM‐17 treatment regimen. Bortezomib administered intravenous at days 1, 4, and 7 (48 hours pretreatment to deplete MGMT protein) before target TMZ 200 mg/m^2^ dose for 5 days (from and including days 3‐7), repeated in six cycles. In *n* = 3 patients per each dose 150 vs 175 vs 200 mg/m^2^ TMZ in dose pathfinding, safety evaluation. Clinical chemistry for renal, hepatic, and bone marrow monitoring for toxicity based on CTCAE v. 4.03. MRI tumor monitoring radiological response assessment based on RANO criteria. Rationale for sequential treatment schedule based on preclinical data. B, sequential administration in BORTEM‐17 clinical trial vs (C) previous studies where BTZ on days 1, 4, 8, and 11 was given concomitantly with TMZ from day 1 to 5, when MGMT levels were high. Dashed boxes mark days when TMZ doses might be more effective, (B) all five doses vs (C) three doses every month. BTZ, bortezomib; CBC Diff, complete blood count with differential test; CMP, comprehensive metabolic panel; KPS, Karnofsky performance score; LFT, liver function test; MGMT, O^6^‐methyl guanine DNA methyltransferase; MRI, magnetic resonance imaging; NANO, neurologic assessment in neuro‐oncology; QoL, quality of life; TMZ, temozolomide

The study protocol (Figure 1A) was approved by the regional ethical board for Western Norway (2017/2084) and the Norwegian Medicines Agency (17/17445‐17). All eligible patients signed the approved consent form for study participation before undergoing any study related procedures. Inclusion/exclusion criteria are summarized in the Supporting Information and further details available on: https://clinicaltrials.gov/ct2/show/NCT03643549?term=Bortezomib+and+temozolomide&cond=GBM&draw=2&rank=1

Ten patients (*n* = 8 males and *n* = 2 females) were enrolled between September 2018 and October 2019 and their characteristics are summarized in Table 1. BTZ 1.3 mg/m^2^ was administered as 30 seconds bolus intravenous injection on days 1, 4, and 7 during each 4th week chemotherapy cycle. The first three patients received 150 mg/m^2^ TMZ per oral 5 days/wk starting on day 3 after BTZ administration in the first cycle and thereafter 175 and 200 mg/m^2^ every 4 weeks in cycles 2 and 3, respectively (Figure 1A). If all three patients at a given TMZ dose did not develop a dose limiting toxicity (ie, grade 3 or 4 on Common Terminology Criteria for Adverse Events [CTCAE]), the next cohort of three patients would be treated at the next TMZ dose level. The ultimate aim was to attain combination treatment with BTZ and TMZ at 200 mg/m^2^ which was closest to the most effective dose in the preclinical study.^22^ Total treatment duration per patient was estimated for 6 to 12 months unless disease progression.

**Table 1:** BORTEM‐17 patient characteristics

Patient ID	Age	Sex	MGMT status	% of methylation (pyrosequence) mean ± SEM	KPS	Molecular pathology				No. of recurrence before recruitment	Treatment after recurrence before recruitment	Treatment after withdrawn	No. of BORTEM cycle after recruitment	OS, mo	Survival after first recurrence	Survival after 2nd recurrence	Survival after recruitment
						KIR2DS4	IDH1	ATRX	TP53								
P01	45	M	UM	3 ± 1.1	90	Func/Del	Mut	Mut	Mut	2	SRS + TMZ	LAVA	3	27.3	13.4	7.1	6.6
P02	53	F	UM	2 ± 0.6	80	Func/Func	Wt	Wt	n/a	2	S + SRS	No antineoplastic treatment	2	24.0	9.6	6.7	6.2
P03	54	M	UM	2 ± 0.6	80	Del/Del	Wt	n/a	n/a	2	TMZ	Lomustin + RT	6	33.8	18.6	15.2	14.5
P04	40	F	UM	3 ± 1.7	90	Del/Del	Mut	Wt	Wt	1	No antineoplastic treatment	No antineoplastic treatment	2	13.8	4.7	2.1	3.8
P05^a^	35	M	UM	2 ± 0.3	90	Del/Del	Mut	Mut	Mut	2	S	LAVA	2	30.6	25.0	14.7	14.4
P06	35	F	UM	3 ± 0.7	90	Func/Func	Wt	Wt	Wt	2	SRS	SRS	2	15.2	6.1	4.9	3.2
P07^a^	26	M	UM	1 ± 0.2	100	Del/Del	Mut	Mut	Wt	1	No antineoplastic treatment	LAVA	2	28.0	11.3	8.9	10.6
P08	57	M	UM	2 ± 0.4	80	Func/Del	Wt	Wt	n/a	1	No antineoplastic treatment	LAVA	2	18.7	4.6	2.3	4.2
P09	58	M	UM	2 ± 0.8	90	Del/Del	Wt	n/a	n/a	2	SRS	No antineoplastic treatment	2	12.8	3.9	2.6	2.2
P10	52	M	UM	4 ± 1.3	80	Func/Del	Wt	n/a	n/a	2	S	No antineoplastic treatment	2	11.2	6.4	3.3	2.4
Median survival, mo														21.4	8.0	5.8	5.2

Abbreviations: ATRX, α‐thalassemia/mental retardation, X‐linked; BORTEM‐17, bortezomib and temozolomide trial 2017; Del, KIR2DS4*00101 deleted (Del/Del homozygous); F, female; Func, KIR2DS4*00101 full length (Func/Func homozygous, Func/Del heterozygous); IDH1, isocitrate dehydrogenase‐1; KIR2DS4, killer cell immunoglobulin like receptor, two Ig domains and short cytoplasmic tail 4; KPS, Karnofsky performance status; LAVA, combination of lomustine, avastin and vincristine; M, male; Mut, mutated; n/a, not available; OS, (overall survival in months) from date of first operation; RT, radiotherapy; S, surgery; SRS, stereotactic radio surgery; Survival after first recurrence, survival after date of MRI showing 1st progression; Survival after recruitment, survival after date of recruitment to last date of update; Survival after second recurrence, survival after date of MRI showing 2nd progression; TMZ, temozolomide; TP53, tumour protein 53; UM *MGMT*, unmethylated O^6^ methylguanine DNA methyltransferase; Wt, wild type.

^a^ Alive.

### 2.2 NeuroImaging

Brain magnetic resonance imaging (MRI) was performed using the 3T Siemens MAGNETOM Prisma (Siemens Healthineers, Munich, Germany) at the Radiology Department at Haukeland University Hospital (details of sequences and quantification of radiologic tumor growth are reported in the Supporting Information). MRI was undertaken no earlier than 2 weeks before enrollment to confirm disease progression using T2 and T1 MRI sequences with and without gadolinium contrast agent (Clariscan; GE Healthcare) and reported by the study neuroradiologist based on RANO criteria.^25^ MRI was also undertaken at day 56, after two cycles of BTZ and TMZ treatment, and thereafter every 8th week until endpoint evaluation at 6 months.

### 2.3 Clinical evaluation and *self‐reported quality of life*

Clinical evaluation including neurological assessment according to Neuro‐Oncology (NANO) scale,^26^ EQ‐5D‐5L quality of life questionnaire,^27^ and Karnofsky performance status (KPS) were recorded during consultations every 4th week and before MRI evaluations. Hematological tests were obtained within 1 week of study enrollment and subsequently within each treatment cycle on days 1, 4, 7, 11, 15, 22, and 28 before commencing the next cycle. Molecular pathology including *MGMT* promoter methylation, *IDH1, TP53*, and *ATRX* mutation analysis was undertaken at the pathology department. Adverse events were graded using the National Cancer Institute CTCAE version 4.03.

### 2.4 Immune monitoring

Immune monitoring was performed on peripheral blood mononuclear cells (PBMCs) before, during and after BTZ + TMZ treatment in time course analyses on day 1, 4, 7, 11, 15, and 22 of the first treatment cycle. PBMCs were stained with fluorescent conjugated primary antibody mix for NK and T cells (Table S1 and Figure S1), data acquired on BD LSR FORTESSA (BD Biosciences, Trondheim, Norway) and analysed with FlowJo software version 10 (Tree Star Inc, Ashland, OR) as detailed in the Supporting Information.

### 2.5 Statistical analysis

Linear mixed effects model regression analysis was used to analyse tumor volumes, EQ‐5D‐5L health scores and their correlation with NANO scores and ability to predict KPS throughout the course of treatment using Stata version 15.1 (StataCorp LLC, Texas). One‐ and two‐way analysis of variance (ANOVA) with Bonferroni correction for multiple testing was used to analyse immunomonitoring and enzyme‐linked immunosorbent assay data using Graphpad PRISM 8.0 software (La Jolla, CA). Two‐sided *P* values less than .05 were considered significant.

## 3 RESULTS

### 3.1 BORTEM‐17 trial schedule

Our treatment regimen was based on a previous preclinical study where we demonstrated that BTZ pretreatment of GBM cells for 48 hours depleted MGMT levels and sensitized to TMZ. Thus, the sequential schedule was adopted, administering intravenous bolus 1.3 mg/m^2^ BTZ on days 1, 4, and 7 before target TMZ 200 mg/m^2^ dose for 5 days (starting from and including days 3‐7), repeated in six cycles (Figure 1A). Previous studies had administered BTZ on days 1, 4, 8, and 11, where this was concomitantly administered with TMZ from day 1 to 5 (when MGMT levels would be high the first 2 days in *MGMT* unmethylated patients). Our rationale was thus to maximize the therapeutic potential of all five TMZ doses by only administering the chemotherapy 48 hours after BTZ when MGMT levels would be lowest (Figure 1B,C).

### 3.2 BORTEM‐17 patient characteristics

The basic clinical and molecular characteristics of the patients are indicated in Table 1. Our patient cohort included eight males and two females with a median age of 49 years (range, 26‐58) and median KPS score 80 (range, 80‐100) at enrollment. In accordance with the inclusion criteria, all patients had unmethylated *MGMT* promoter (quantitatively <4% methylation), while *n* = 4/10 (40%) were *IDH1* R132H mutant with accompanying *TP53* and *ATRX* mutations. Five patients possessed the KIR2DS4*00101 allele as homozygous or heterozygous alleles that we previously demonstrated to be an independent good prognostic factor.^28^ At the time of primary diagnosis, five patients had undergone gross total resection while the remaining five had subtotal resection or biopsy. Primary tumor location was temporal (2/10), frontal (3/10), parietal lobes (3/10), and in two patients multifocal. Seven patients (70%) were enrolled onto the study protocol after their second recurrence and three of them had had secondary surgery. Three patients were treated at first tumor recurrence.

### 3.3 BTZ administered in combination with TMZ is appropriately metabolized

**Figure 2:**
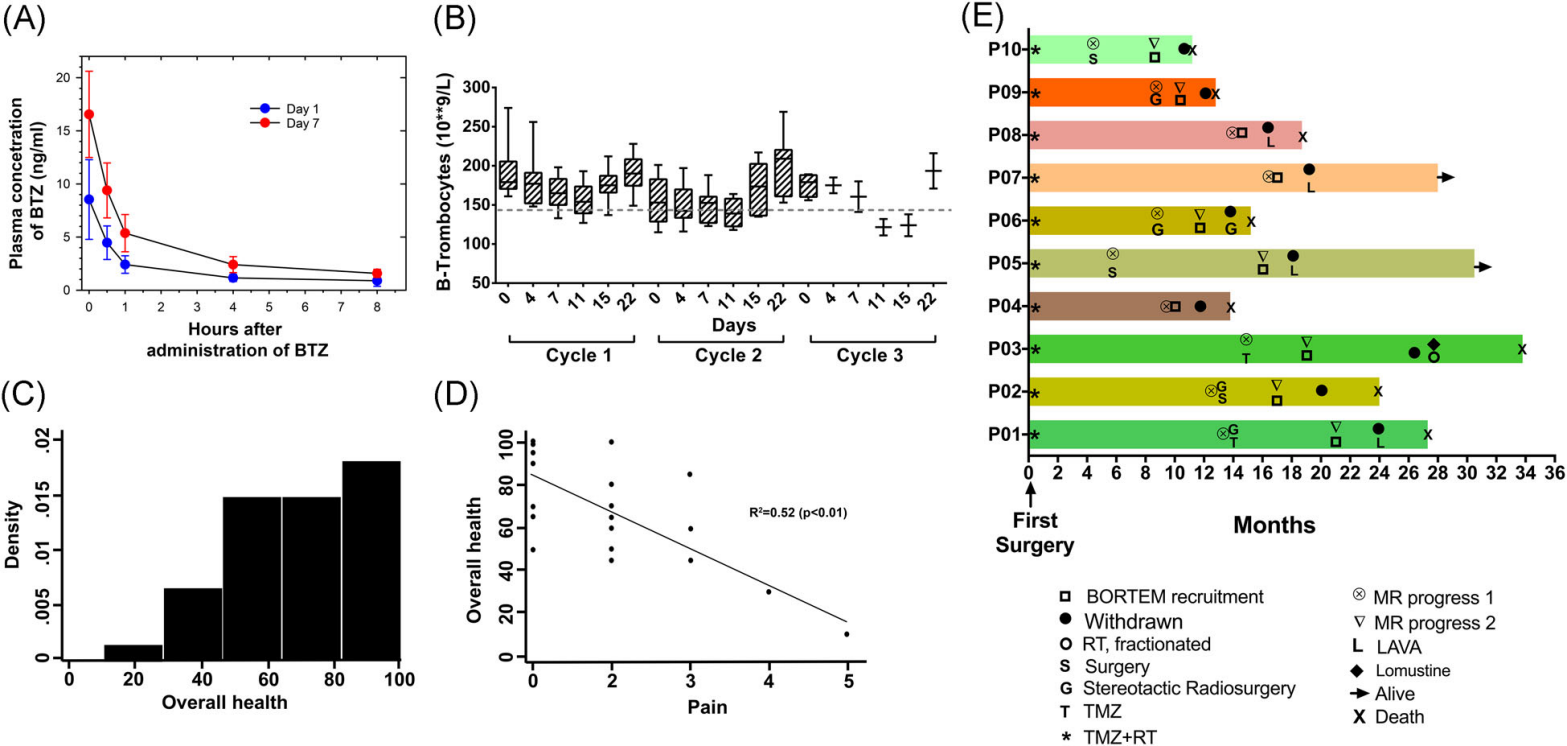
Clinical, radiological and quality of life response. A, Pharmacokinetic analysis of bortezomib concentration (ng/mL) in plasma of patients following five timepoints on day 1 and 7 of treatment. B, Thrombocyte counts during the first three treatment cycles. Data represent the mean ± SEM from *n* = 10 patients. C, Density scores for perception of overall health on EQ‐5D‐5L questionnaire. D, Correlation of self‐reported overall health and levels of pain on EQ‐5D‐5L. E, Swimmer plot of individual patients depicting treatment start/stop times of all trial patients, aligned according to their first surgery. Bars represent survival in months from first surgery, first and second MRI progression. Rightward arrow indicates that the patient was still alive at the time of final data collection

Next, we investigated time course pharmacokinetic distribution of BTZ after administration to establish that in these heavily pretreated GBM patients, BTZ was metabolized as previously described.^29^ The plasma concentration of BTZ showed a biphasic decline, with a fast distribution phase and a slower elimination phase consistent with a two‐compartment model (Figure 2A). There was also an increase in plasma concentrations at all time‐points on day 7 where estimated *C*_max_ was twice as high, (*P* < .001; Figure 2A and Table 2) compared to day 1. Both the increased *C*_max_ and area under the curve values (ng/mL) were consistent with previous findings in adult patients receiving BTZ on days 1, 4, 8, and 11.^30^ Taken together, the data indicate that BTZ was metabolized similarly to chemotherapy naïve patients.

**Table 2:** Estimated bortezomib concentration in plasma at day 1 and 7 in patients receiving BTZ at day 1, 4, and 7

	Day 1		Day 7	
Patient ID	*C*_max_	AUC	*C*_max_	AUC
P01	6.10	460.2	19.35	2403.7
P02	5.56	1178.2	16.17	2845.3
P03	15.47	2344.6	15.14	2071.0
P04	6.37	889.8	14.52	1883.7
P05	7.76	1068.3	16.05	2538.4
P06	4.43	1096.9	10.97	2189.9
P07	5.51	1125.2	14.19	1818.6
P08	9.42	1403.1	14.39	2738.8
P09	13.37	1513.8	18.80	2985.6
P10	11.37	2159.9	25.90	4015.3
Average	8.54	1324.0	16.55***	2549.0***
STD	3.748	701.90	4.059	380.43

*Note*: Plasma concentration at *t* = 0 hour (C_max_) and AUC at day 1 and 7 in 10 patients receiving BTZ at day 1, 4, and 7.

Abbreviation: AUC, area under the curve; STD, standard deviation

*** Denotes significance at *P* < .001, respectively, using two‐sided Student's *t* test to compare mean *C*_max_ or AUC from day 1 and 7.

### 3.4 Combination BTZ and TMZ is well tolerated

Sequential therapy with BTZ and TMZ was safe, well tolerated and thrombocyte levels consistently normalized by day 22 of each cycle (Figure 2B and Table 3). There was no dose limiting toxicity and most adverse events were mild to moderate in severity (grades 1 or 2). Only 6/10 patients reported toxicities that included grade 2 fatigue (3/10 patients: 30%) where in one patient it was worsening of pre‐existing fatigue. Grade 2 pruritus and urticaria was experienced by 1/10 patient (10%), while grade 1 paresthesia, nausea, gastrointestinal infection, intracranial hemorrhage, and diarrhea/constipation were recorded in 5/10 (50%) patients, respectively (Table 3).

**Table 3:** Observed adverse events

	Any grade		Grade 1		Grade 2	
Adverse events	*n*	%	*n*	%	*n*	%
Fatigue	3/10	**30**	0	**0**	3/10	**30**
Nausea	1/10	**10**	1/10	**10**	0	**0**
Constipation	1/10	**10**	1/10	**10**	0	**0**
Pruritus	1/10	**10**	0	**0**	1/10	**10**
Paresthesia	1/10	**10**	1/10	**10**	0	**0**
Urticaria	1/10	**10**	0	**0**	1/10	**10**
Vomiting	1/10	**10**	1/10	**0**	0	**0**
Gastrointestinal infection (suspected)	1/10	**10**	1/10	**0**	0	**0**
Intracranial hemorrhage	1/10	**10**	1/10	**0**	0	**0**
Diarrhea	1/10	**10**	1/10	**0**	0	**0**
Thrombocytes	8/10	80	8/10	80	0	0
Total number of patient with adverse events	**6/10**	**60**	**5/10**	**50**	**4/10**	**40**
Adverse events due to BTZ treatment	3/10	**30**	2/10	**20**	1/10	**10**
Adverse events due to TMZ treatment	5/10	**50**	1/10	**10**	4/10	**40**
Worsening of a pre‐existing condition	2/10	**20**	1/10	**10**	1/10	**10**

*Note*: Bold values indicate percentage.

### 3.5 Pain had greatest impact on self‐reported quality of life

We used the EQ‐5D‐5L questionnaire to detect clinically relevant differences in health‐related quality of life and sought to identify variables that most predicted the patients' general quality of life. The patients' overall health evaluations were skewed towards higher values indicating generally good perceived health (Figure 2C). However, unadjusted analyses showed a significant relation between overall health perception and usual activities of daily living (*b*‐coefficient, −18.26; 95% confidence interval [CI] [−30.904 to −5.020]; *P* = .005). An increase of pain perception by one point on the EQ‐5D‐5L was associated with 17 times reduced overall health assessment (*b*‐coefficient, −17.592; 95% CI [−24.909 to −10.276]; *P* < .001). Likewise, perceived increased anxiety or depression was greatly correlated with diminished overall health assessment (*b*‐coefficient, −13.2048; 95% CI [−22.842 to −3.254]; *P* = .009). We next performed a stepwise analysis to determine which variable had the most significant impact on patients' overall health perception and found that pain (*b*‐coefficient, −13.293; 95% CI [−18.567 to −8.019]; *P* < .001) and ability to perform usual activities (*b*‐coefficient, −11.309; 95% CI [−15.090 to −7.528]; *P* < .001) had the most impact (Figure 2D). However, the time point of the patients' pain experience was not significant (*b*‐coefficient, 1.628; 95% CI [0.002‐3.255]; *P* = .050) indicating that this was not associated with their study participation. Moreover, analysis of individual effects of mobility (*P* = .001), self‐care (*P* = .003), ability to perform usual daily activities (*P* = .001), pain (*P* < .0001), depression/anxiety (*P* = .012) all showed inverse association with KPS. Median KPS after second evaluation was 80 (range, 70‐100) and 80 (range, 60‐90) at the third evaluation. In stepwise adjusted analyses, self‐care (*b*‐coefficient, −4.893; 95% CI [−9.654 to −0.132]; *P* = .044) and ability to perform usual daily activities (b‐coefficient, −4.39; 95% CI [−6.967 to −1.826]; *P* = .001) were most correlated with KPS. Furthermore, stepwise adjusted correlation analyses of NANO scale variables with KPS, revealed inverted association of vision (*P* = .020), strength (*P* < .0001), ataxia (*P* < .0001), and language (*P* = .010) with KPS, however, ultimately, the ability to perform usual daily activities (*b*‐coefficient, −3.587; 95% CI [−5.534 to −1.640]; *P* < .0001), ataxia (*b*‐coefficient, −14.03; 95% CI [−21.554 to −6.514]; *P* < .0001), and strength (*b*‐coefficient, −8.587; 95% CI [−14.896 to −2.278]; *P* = .008) had the greatest effect on KPS.

### 3.6 Clinical and radiological responses

**Figure 3:**
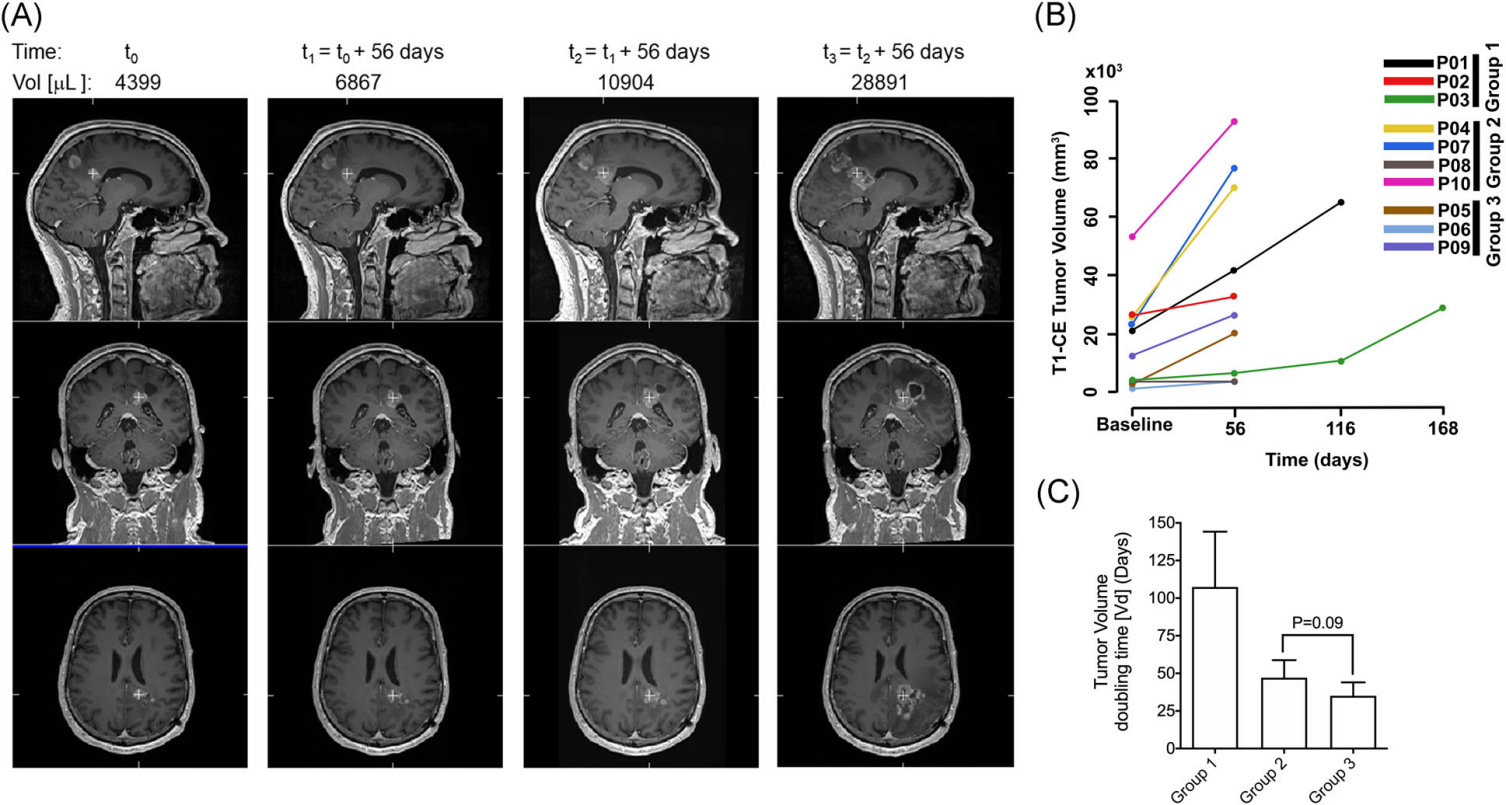
Tumour growth and population doubling time. A, Coregistered 3D T1 weighted gadolinium contrast enhanced serial MR image of patient‐03 (*IDH*wt; *MGMT* UM, 54 years male) treated for 6 months, time indicated in days and tumour volume in µL. B, Mean of 3D measured tumour volumes in mm^3^ from T1‐weighted MR images with contrast of all patients, and (C) tumor population doubling time (in days) for group 1, 2, and 3 patients

As of April 2020, 10 patients met the RANO radiological or clinical progression criteria, eight were withdrawn after two cycles. Two patients completed three and six cycles of treatment. Of these 10 patients, eight died of tumor progression while on second line treatment or after study medication was discontinued (Figure 2E and Table 1). After study treatment withdrawal five patients (P01, P03, P05, P07, P08) were transitioned to lomustine or the combination lomustine, avastin and vincristine (LAVA), whereas four patients did not receive further anti‐neoplastic treatment, (P02, P04, P09, P10; Table 1). Notwithstanding, patient‐02 was alive for 4.5 months after study withdrawal without further anti‐neoplastic treatment, before his death. Patient‐03 had radiological stable disease according to RANO criteria after the second cycle and completed the six cycles of treatment (Figures 2E and 3A). After a treatment break, he had a third MRI progression, and received reirradiation and lomustin and continued lomustine every 6th week. He died 14.5 months after inclusion in the BORTEM‐17 study and had overall survival of 33.8 months. Patient‐05 and ‐07 are still alive and have survived 14.4 and 10.6 months, respectively, post recruitment. No problems of noncompliance were recorded. After recruitment, median survival of the 10 patients was 5.2 months and median overall survival was 21.4 months (Table 1).

### 3.7 Slower tumour growth kinetics in group 1 patients with positive MRI and clinical responses

The patients were grouped a priori based on clinical or MRI progression, where group 1 patients (01, 02, and 03) were characterized by long survival, stable clinical symptoms and/or MRI confirmed stable disease during BORTEM‐17 treatment. Group 2 patients (04, 07, 08, and 10) exhibited rapid clinical deterioration and MRI confirmed progressive disease, while group 3 patients (05, 06, and 09) exhibited mixed positive and negative clinical and radiological effects that did not fit the profiles of neither group 1 nor group 2. The median TMZ dose received for group 1, 2, and 3 patients was 150, 175, and 175 mg/m^2^, respectively. Patient‐03 from group 1 had a left splenium contrast enhancing lesion and achieved clinical and radiological stable disease (20% progression from baseline according to RANO criteria) 56 days after commencing treatment (Figure 3A), subsequently completing all six therapy cycles. However, volumetric quantification of contrast enhancing tumour growth on 3D T1 weighted MRI revealed overall change of growth over time in all patients (*b*‐coefficient, 12 403.7; 95% CI [4880.8‐19 926.7]; *P* = .001; Figure 3B). Mean‐ tumour volumes between the groups were not statistically different, although compared to baseline volumes, there was significant change after 112 days (b‐coefficient, 24 220; 95% CI [−6435.8‐42 004.2]; *P* = .008) and 168 days (*b*‐coefficient, 30 828.4; 95% CI [7512.3‐54 144.6]; *P* = .01) in group 1 patients. Likewise, group 2 patients' MRI‐tumour volumes after 56 days were significantly different from group 1 baseline volumes, (*b*‐coefficient, 43 825.6; 95% CI [14 670.8‐72 980.4]; *P* = .003). Group 1 patient tumours exhibited the slowest growth rate indicated by longest population doubling time (approximately 107.4 days) compared to group 2 and 3 patient tumours (47.1 and 35.1 days, respectively; Figure 3C).

### 3.8 Immunological mechanisms of patients with positive clinical responses

BTZ‐induced tumour death generates immunogenic antigens and sensitizes to dendritic cell immunotherapy.^24^, ^31^, ^32^ Thus, we investigated the patients' T cell activation and maturation status in blood and cytokines circulating in their plasma at baseline, after exposure to BTZ only (within the first 8 hours after administration), BTZ + TMZ (on days 4‐7) and during the 3 weeks recovery period (days 11, 15, and 22) in cycle 1. We also investigated responses to stimulation with PMA/ionomycin or autologous tumour cells ex vivo of patients' PBMCs obtained at baseline (BTZ naïve) versus those obtained during recovery (day 22‐56) after initial exposure to BTZ + TMZ.

**Figure 4:**
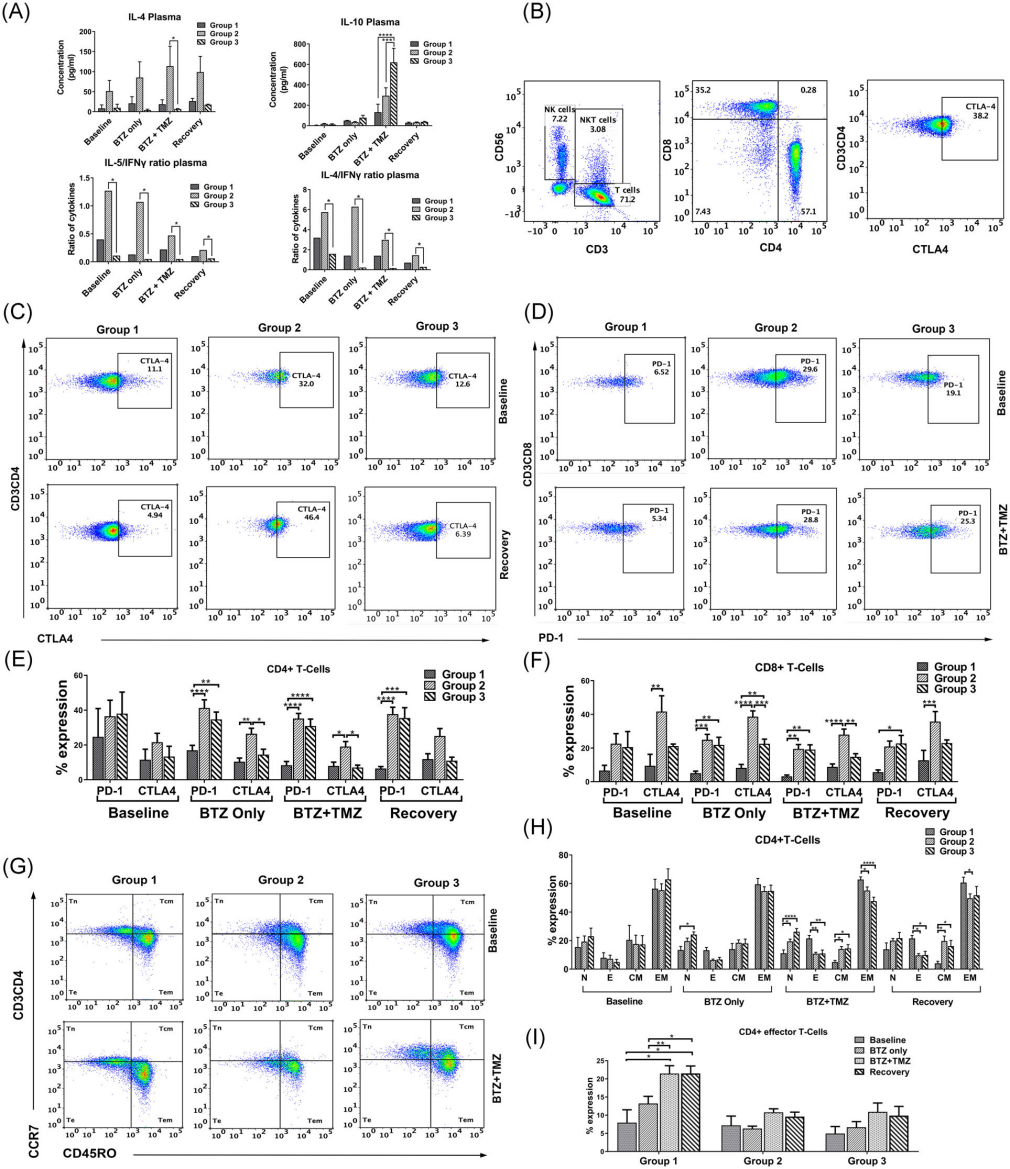
Immune mechanisms and augmented differentiation of CD4^*+*^ and CD8^*+*^ T cell subsets in patients with positive clinical responses. A, Cytokine concentrations in pg/mL in patients' plasma, (top panels) IL‐4 and IL‐10, and (bottom panels) the ratio between the average concentrations of IL‐5 and IL‐4 against IFNγ during treatment. B, Representative dot plots showing (from left to right) patient NK and T cell lymphocytes in CD56 vs CD3 gates, CD8^+^ vs CD4^+^ T cell subsets and CD3^+^CD4^+^ T cells expressing CTLA‐4. C, Representative dot plots showing CD3^+^CD4^+^ T cell subsets expressing CTLA‐4 and (D) Representative dot plots showing CD3^+^CD8^+^ T cell subsets expressing PD‐1. E, Mean ± SEM % of CD3^+^CD4^+^ T cells expression of PD‐1 and CTLA‐4 in group 1, 2, and 3 patients before, during and after treatment with BTZ and TMZ. F, Mean ± SEM % of CD3^+^CD8^+^ expressing PD‐1 and CTLA‐4 before and after treatment with BTZ and TMZ. G, Dot plots showing CCR7 vs CD45RO within CD3^+^CD4^+^ T cell subsets in group 1, 2, and 3 patients, before and during treatment with BTZ and TMZ. H, Mean ± SEM % expression of naïve (N), effector (E), central memory (CM) and effector memory (EM) CD4^+^ T‐cells, and (I) CD4^+^ T‐effector cells before, during and after treatment with BTZ and TMZ in group 1, 2, and 3 patients. *P < .05, **P < .01, ***P < .001, ****P < .0001, *n* = 10 patients. BTZ, bortezomib; IFNγ, interferon γ; IL, interleukin; NK, natural killer; TMZ, temozolomide

Remarkably, group 2 patients had substantially higher levels of multiple immune tolerising Th2 cytokines in plasma compared to group 1 and 3 patients. During BTZ + TMZ treatment fourfold to sevenfold higher concentrations of interleukin 4 (IL‐4) was observed in group 2 compared to group 3 patients (*P* < .05; Figure 4A). Group 2 patients also had consistently higher IL‐5/IFNγ and IL‐4/IFNγ ratios (*P* < .05) from baseline throughout treatment compared to group 3 patients. In contrast, group 3 patients had threefold higher IL‐10 plasma levels compared to group 1 and group 2 patients during BTZ and TMZ treatment (two way ANOVA, *P* < .0001 and *P* < .001, respectively; Figure 4A), implying a tolerising effect of TMZ chemotherapy through IL‐10, in particular for group 3 patients.

### 3.9 Rapidly progressing patients are characterized by Th2 immune responses and tolerised CD8^+^ T cell phenotype

Given their profound Th2‐cytokine driven anti‐inflammatory responses, we investigated their relative expression of cytotoxic T‐lymphocyte–associated antigen 4 (CTLA‐4) and programmed death 1 (PD‐1) immune checkpoints on CD4^+^ and CD8^+^ T cell subsets (Figure 4B‐F) as an indication of negative immune regulation and T cell exhaustion. Although there were no significant differences in numbers of CD4^+^CTLA‐4^+^ T cells at baseline (Figures 4C and 4E), CD4^+^CTLA‐4^+^ T cells were increased by twofold to threefold after BTZ and during combination with BTZ and TMZ in the rapidly progressing group 2 patients compared to group 1, (Figures 4C and 4E; one way ANOVA, *P* < .01, *P* < .05) respectively, and compared to group 3 patients, (*P* < .05, respectively). PD‐1^+^ CD4^+^ T cells were increased by twofold to fourfold in group 2 compared to group 1 patients during combination BTZ with TMZ and later, during recovery (one way ANOVA, *P* < .0001, both, Figures 4C and 4E). Group 3 patients also upregulated numbers of PD‐1^+^ CD4^+^ T cells by twofold to fourfold compared to group 1 patients (one way ANOVA, *P* < .0001, Figures 4C and 4E) during combination BTZ with TMZ when IL‐10 levels were elevated in their plasma (Figure 4A) and in the recovery period, respectively.

Larger fractions of cytotoxic CD8^+^ T cells from group 2 patients expressed CTLA‐4 compared to group 1 patients at baseline (one way ANOVA, *P* < .01), during BTZ (*P* < .0001), also after combination BTZ with TMZ treatment (one way ANOVA, *P* < .0001) and during recovery, (*P* < .001; Figure 4F). Group 2 patients also had higher levels of CD8^+^ CTLA‐4^+^ T cells compared to group 3 patients during BTZ (one way ANOVA, *P* < .01), at combination BTZ with TMZ treatment (*P* < .01; Figure 4F). Collectively, these data might indicate that CD4^+^ T cells from the rapidly progressing patients are biased towards a Th2 phenotype and tolerised at earlier stages (days 1‐7 of BTZ, as well as BTZ + TMZ treatment). This is consistent with the profound Th2 cytokine profiles in their plasma. On the other hand, CD8^+^ effector T cells from rapidly progressive and mixed benefit patients from group 2 and 3, respectively, upregulated the inhibitory receptor PD‐1 (Figures 4D and 4F) only at later stages during recovery (days 4‐15.) Importantly, this phenomenon was not evident in group 1 patients.

### 3.10 Augmented differentiation of CD4^+^ and CD8^+^ T cell subsets in patients with positive clinical responses

Given the potential tolerization and exhaustion of T cells denoted by differential Th2/Th1 cytokine ratios and expression of PD‐1 and CTLA‐4 checkpoint molecules in the patients undergoing treatment, we sought to investigate their maturation phenotypes. We used CCR7, CD45RO, CD28 surface expression to discriminate distinct differentiation stages from naïve (T_N_), effector (T_E_), central memory (T_CM_), effector memory (T_EM_), and NK‐cells (Figures 4G and S2). While there was no difference in differentiated CD4^+^ T cell subsets between patients at baseline, group 1 patients with positive clinical and immunological responses had increased CD4^+^ T_E_ subsets after BTZ + TMZ combination treatment compared to rapidly progressive group 2 and 3 patients (two way ANOVA, *P* < .001, respectively (Figure 4G,H) and during the recovery period (two way ANOVA *P* < .05; Figure 4G,H). Importantly, CD4^+^ T_E_ subsets were not only increased after BTZ + TMZ treatment within group 1 patients, but also during recovery compared to baseline (two way ANOVA, *P* < .05, both), and BTZ only treatment, (Figure 4I; two way ANOVA *P* < .01 and *P* < .05, respectively). Correspondingly, these patients had fewer CD4^+^ T_CM_ during combination BTZ + TMZ treatment and recovery periods (one way ANOVA, *P* < .05, and *P* < .01), respectively (Figure 4H). These data indicate that the group 1 patients with positive clinical and Th1 driven immune responses possibly mobilized and expanded CD4^+^ T_E_ subsets in response to novel antigens produced as a result of BTZ + TMZ induced tumor cell death.

### 3.11 Patients with positive clinical responses diminish CTLA‐4^+^ CD4^+^T cells and attenuate IL‐10 secretion after treatment

**Figure 5:**
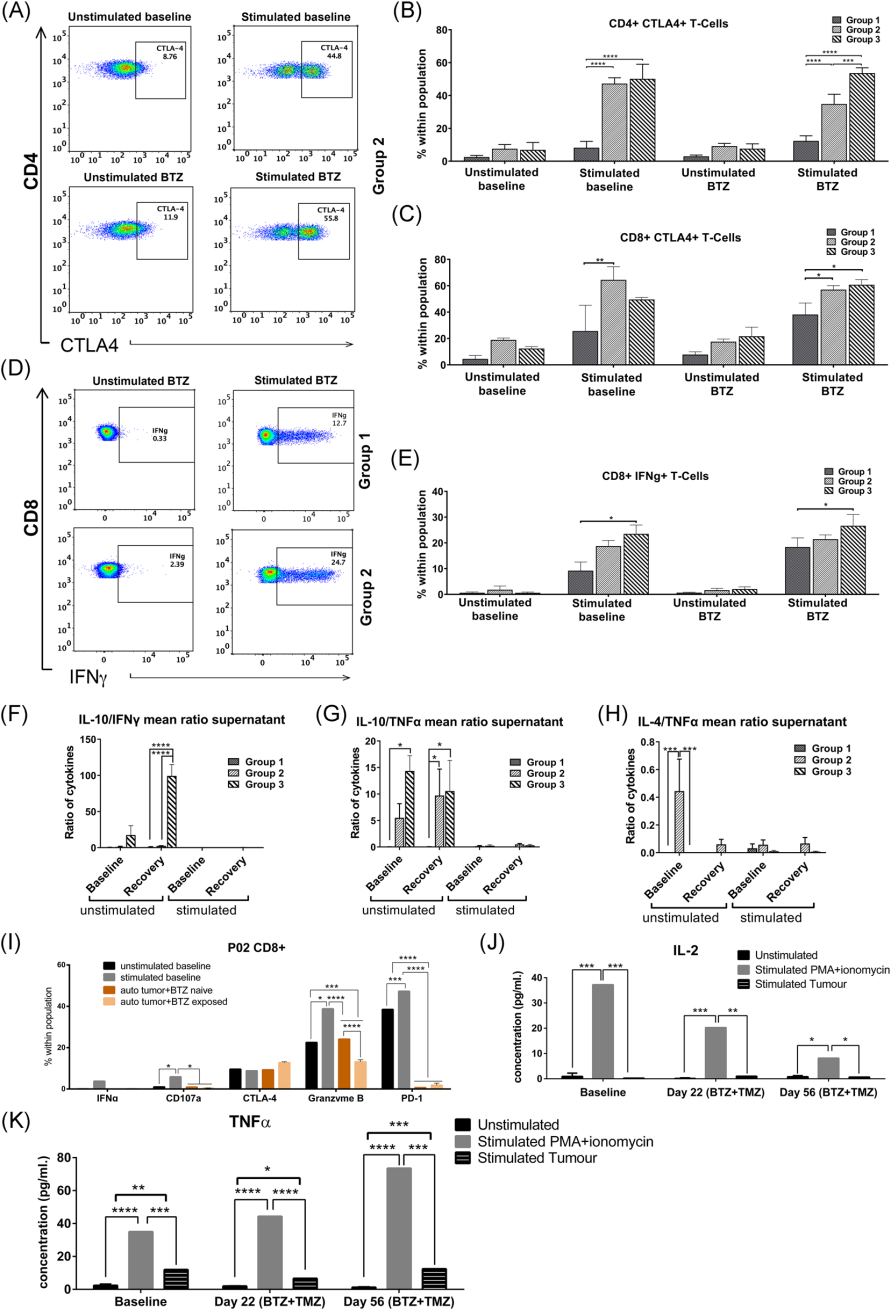
PBMCs from patients with positive clinical responses diminish CTLA‐4^*+*^ CD4^*+*^Th cells, attenuate IL‐10 secretion and have moderate intracellular IFNγ after bortezomib + temozolomide treatment. A, Representative dot plots showing CD4 vs CTLA‐4 in patient PBMCs unstimulated and stimulated, with PMA/ionomycin before or after exposure to BTZ or BTZ + TMZ treatment in situ. B, Mean ± SEM % of CD4^+^ CTLA‐4^+^ T cells at baseline or after treatment/stimulation conditions in group 1, 2, and 3 patients. C, Mean ± SEM % of CD8^+^ CTLA‐4^+^ at baseline or after treatment/stimulation conditions in group 1, 2, and 3 patients. D Representative dot plots showing intracellular IFNγ in CD8^+^T cells in patients' PBMCs unstimulated and stimulated with PMA/ionomycin in group 1 vs 2 patients. E, Mean ± SEM % of CD8^+^ IFNγ^+^ T cells at baseline or after treatment/stimulation conditions in group 1, 2, and 3 patients. Cytokines present in supernatants from patients' PBMCs after stimulation with PMA/ionomycin represented as mean ± SEM of Th2/Th1 ratio between (F) IL‐10 and IFNγ, (G) IL‐10 and TNF‐α, and (H) IL‐4 and TNF‐α in group 1, 2, and 3 patients at baseline or during treatment recovery. I, Mean ± SEM % expression of markers in patient‐02 tumour or PBMCs unstimulated and stimulated with PMA/ionomycin or P02‐tumour before or after exposure to bortezomib + temozolomide in situ. Cytokine concentrations in pg/mL from supernatants from patient‐02 tumour or PBMCs unstimulated or stimulated ex vivo with PMA + ionomycin or patient‐02 tumour or before and after exposure to bortezomib + temozolomide in situ of (J) IL‐2 and (K) TNF‐α. *P < .05; **P < .01; ***P < .001: ****P < .0001, *n* = 10 patients. BTZ, bortezomib; IFNγ, interferon γ; IL, interleukin; TMZ, temozolomide; TNF‐α, tumor necrosis factor α

To investigate whether the Th2‐biased and tolerised T cell phenotype before and after BTZ treatment in some patients could be reversed, we used PMA/ionomycin to stimulate PBMCs isolated from patients' blood at baseline and on day 22 during recovery after exposure to BTZ and TMZ therapy. Stimulation of baseline PBMCs with PMA/ionomycin increased the level of CTLA‐4^+^CD4^+^ Th cells in group 2 and 3 patients by sixfold compared to group 1 patients (Figure 5A,B, two way ANOVA, *P* < .0001, respectively), and CTLA‐4^+^CD8^+^ T cells by threefold (group 2 vs 1, *P* < .001; Figure 5C). However, stimulation of PBMCs that had been exposed to BTZ + TMZ treatment in situ induced the greatest numbers of CTLA‐4^+^ expressing CD4^+^ Th cells in group 3, compared to both group 1 and 2 patients (Figure 5B, two way ANOVA *P* < .0001), respectively. Notably, stimulation of PBMCs from rapidly progressing group 2 patients exposed to BTZ + TMZ treatment diminished the fractions of CTLA‐4^+^ CD4^+^ Th cells by ca. 15% (Figure 5B) compared to stimulation at baseline, implying positive effects of the treatment, also indicated by diminishing immune suppressive Th2/Th1 cytokine ratios in plasma after treatment (Figure 4A). CD8^+^ T cell subsets from group 1 patients had moderate levels of intracellular interferon γ (IFNγ) compared to group 3 patients stimulated at baseline or during treatment recovery (two way ANOVA *P* < .05 respectively, Figure 5D,E). Correspondingly, group 3 patients with greatest fractions of CTLA‐4^+^CD4^+^ Th cells also exhibited greater immunosuppressive IL‐10/IFNγ ratios at recovery compared to both group 1 and group 2 patients (*P* < .0001, respectively; Figure 5F). Confirming the profound immune suppressive and Th2‐driven responses, also observed in plasma cytokines, unstimulated PBMCs from both group 2 and 3 patients exhibited greater IL‐10/TNF‐α at recovery compared to group 1 patients (*P* < .05, respectively; Figure 5G), and IL4/TNF‐α ratios in unstimulated PBMCs at baseline compared to group 1 patients (*P* < .001, respectively; Figure 5H). In patient‐02 from group 1 whose tumour was available for coculture with autologous PBMCs, the CD8^+^T cells were not tolerised by coculture with the tumour, compared to PBMCs not cocultured with tumour cells, or PBMCs stimulated with PMA/ionomycin (one way ANOVA, *P* < .0001, Figure 5I). Robust stimulation with PMA/ionomycin increased CD107a degranulation in CD8^+^ T cells compared to PBMCs exposed or not to BTZ in situ or cocultured with patient‐02' autologous tumour cells (*P* < .05). Nevertheless, release of cytolytic granzyme B by CD8^+^ T cells was least potent upon coculture with autologous tumour cells (*P* < .0001) compared to BTZ naïve PBMCs (Figure 5I). IL‐2 secretion was greatest after stimulation with PMA/ionomycin of PBMCs at baseline and after BTZ exposure (*P* < .001; Figure 5J). Although attenuated, cytotoxic CD8^+^ T cells increased tumour necrosis factor α (TNF‐α) secretion when exposed to autologous patient‐02' tumour cells and when compared to unstimulated PBMCs from baseline (*P* < .01), at day 22 during recovery after BTZ + TMZ treatment (*P* < .05) and at day 56 post treatment (*P* < .001; Figure 5K). Collectively, these data might indicate that this patient's killer T cells were able to launch appropriate responses against his tumour ex vivo.

### 3.12 BTZ induced NK cell maturation phenotype and expression of inhibitory checkpoints

Group 1 patients possessed more mature CD16^+^CD57^+^ NK cells at baseline (*P* < .01), during BTZ (*P* < .05), BTZ +  TMZ treatment (*P* < .01) and during recovery (*P* < .05) compared to group 3 patients, and during BTZ + TMZ (*P* < .05), and recovery (*P* < .0001) compared to group 2 patients (Figure 2A‐2E). Surprisingly, these mature subsets in the group 1 patients upregulated the inhibitory checkpoint receptors NKG2A and PD‐1 (Figure S2F,S2G), and diminished activating DNAM‐1 and CD69 receptors (Figure S2H,S2I). In contrast, during BTZ + TMZ treatment group 2 and 3 patients who secreted high levels of IL‐10 and had less profound T cell responses also decreased CD16^+^CD57^+^ mature NK cell fractions compared to group 1 patients (CD16: group 2 vs 1, *P* < .05; group 3 vs 1, *P* < .01) and CD57: group 2 vs 1, *P* < 0.01; group 3 vs 1, *P* < .05). Within the reduced CD16^+^ CD57^+^ NK cell fractions in group 2 and 3 patients (Figure S2D,S2E) inhibitory NK cells expressing NKG2A and PD‐1 were also diminished compared to group 1 patients (Figure S2F,S2G), whereas subsets expressing DNAM‐1 and CD69 were increased after BTZ and TMZ treatment (Figure S2H,S2I), potentially indicating increased activation.

Taken together, copious IL‐10 levels in plasma may have contributed to greater activation of their NK cells but attenuated T cell responses after BTZ and TMZ treatment. Unfortunately, due to limited PBMCs and lack of access to fresh biopsy tissue for all patients, we could not perform functional assays with purified NK cells to mechanistically interrogate these findings.

## 4 DISCUSSION

We sought herein to investigate as primary objective whether sequential pretreatment with BTZ to sensitize recurrent GBM patients with unmethylated *MGMT* promoter to TMZ chemotherapy was safe and tolerated. This is because previous analyses utilized a concurrent schedule with monotherapy BTZ, metronomic doses of TMZ, or were conducted in newly diagnosed patients.^33^, ^34^, ^35^, ^36^, ^37^ In this phase IB study, we successfully dose escalated TMZ to the target 200 mg/m^2^ dose for combination with 1.3 mg/m^2^ BTZ and no grade 3 or 4 toxicities were registered in our study. This is consistent with previous trials using concurrent schedules^22^ with intensive and prolonged treatment, as well as with various drug combinations.^33^, ^34^, ^35^, ^36^, ^37^, ^38^, ^39^

Our patients scored relatively high on the quality of life EQ‐5D‐5L questionnaire that was previously validated for the Norwegian population.^40^ The ability to perform usual daily activity, ataxia, and changes in strength were the strongest predictors of KPS and this finding is corroborated by a previous study of Norwegian patients after surgery.^41^ BTZ crosses the blood brain barrier in human and mice^22^, ^39^ and higher concentrations were found in brain tissue than in plasma.^39^ Our pharmacokinetic analysis showed similar rapid redistribution of the drug from plasma to tissue as was reported for newly diagnosed patients.^29^, ^30^

Nevertheless, the BORTEM‐17 patients segregated a priori into three groups based on clinical and/or radiological progression. The grouping was concordant with tumor growth characteristics where group 2 patients with the most rapid progression had the greatest tumour burden (*ca*. 34 × 10^3^ mm^3^) at baseline. Moreover, their tumour population doubling time from baseline to first MRI evaluation at day 56 was 47.1 days, nearly twofold faster than the group 1 patients whose baseline tumour volume was ca. 17.2 × 10^3^ mm^3^ and had a prolonged doubling time of 107.4 days. Although two‐thirds patients in groups 1 and 3 had prior treatment with stereotactic radiosurgery (SRS) after their second recurrence and before study recruitment, these patients had differential clinical responses. To minimize the challenge of differentiating tumour recurrence from radiation necrosis, study recruitment was based on MR contrast enhancing lesion greater than or equal to 12 weeks after completed radiotherapy. Furthermore, the SRS treated lesions were not considered as target lesions in the RANO evaluation of tumour progression.

*IDH1* mutant genotype is associated with improved outcome^10^, ^42^ but 2/3 (66%) of best performing group 1 patients had *wt IDH1* compared to 2/4 (50%) and 2/3 (66%) patients from group 2 and 3, respectively. Since the *IDH*‐status had similar heterogeneous frequency within the groups, this was therefore not considered the underlying cause for differential responses of the patients. Furthermore, nine of the 10 patients had de novo GBM, with no evidence of a less malignant lesion before GBM diagnosis. Patient 07's *IDH1*‐mutant GBM tumor had progressed from an initial grade III anaplastic astrocytoma, thus representing a secondary GBM. Thus, the primary vs secondary GBM status also could not explain the differential prognosis between the patient groups. We did not however, classify the tumors based on previously reported gene expression profiles^43^, ^44^, ^45^, ^46^ because we only had access to formalin fixed paraffin embedded tumor tissue. Three molecular GBM subtypes termed proneural, proliferative, and mesenchymal^44^ were identified and reported to have prognostic value that was independent of the World Health Organization histological grading and/or presence of necrosis. Further subclassifications identified four subtypes–classic, mesenchymal, proneural, and neural signatures^45^, ^46^ and now, more recently, greater heterogeneity at the cellular level was confirmed.^43^ However, the proneural GBM subtype with assumed good prognosis in the Phillip et al's^44^ study was associated with poor prognosis in the Verhaak study.^45^ Furthermore, the subtypes coexist and vary spatially within the individual tumors as shown by multiregional sampling. Longitudinal analyses also indicate that the subtype signatures may change over time, affected, for example, by therapy. Thus, although the molecular subtypes provide great insight and opportunities for targeting therapies to the aberrant gene pathways, they fail to demonstrate robust differences in survival between them and are thus, not currently utilized clinically for diagnosis or prognostication. For this reason, we did not classify the tumors according to these molecular subtypes.

Remarkably, group 2 patients who also exhibited profound tolerance within their peripheral immune cell compartments demonstrated by dominant Th2 *vs*. Th1 suppressive cytokine ratios in plasma, consistently elevated PD‐1 and CTLA‐4 immune checkpoints on both CD4^+^ Th and CD8^+^ cytotoxic T cells throughout the treatment. They also had fewer fractions of CD16^+^CD57^+^ mature NK cells and subsets expressing inhibitory NKG2A and PD‐1 checkpoint molecules. In contrast, NK‐ cell populations expressing CD69 and DNAM‐1 were increased after BTZ and TMZ treatment, potentially indicating increased activation. Indeed, when BTZ naïve PBMCs from group 2 patients were stimulated with PMA/ionomycin ex vivo, they responded by secreting suppressive Th2 cytokines such as IL‐4, IL‐10 and upregulated CTLA‐4^+^CD4^+^ and CTLA‐4^+^CD8^+^ T cell fractions. Under the influence of IL‐4, tumor cells secrete IL‐10 which hinders activation and recruitment of adaptive immune mediators to the tumor microenvironment, by for example, downregulating CD28 expression, inhibiting proliferation and secretion of Th1 cytokines (IFNγ, TNF‐α, IL‐2) by T cells, as well as counteracting antigen presentation by dendritic cells.^47^, ^48^ IL‐10 also diminishes tumor cell expression of class I major histocompatibility molecules (MHC),^49^ and provides opportunity for NK cell mediated cytotoxicity. IL‐10 thus also regulates NK cell cytotoxicity and is established to have a dual pro‐ and anti‐inflammatory role in different cell types and under different conditions.^50^ IL‐10 gene polymorphisms that reduce IL‐10 levels correlate with increased melanoma incidence, while high IL‐10 levels are observed at sites of spontaneous rejection in primary melanoma,^51^ potentially NK cell mediated. Intravenous administration of IL‐10 to healthy volunteers induced increased IFN‐γ and granzyme release.^52^ IL‐10 conditioning of NK cells in vitro upregulated mRNA transcripts for type I interferons, high mobility group 1, CD69 and secretogranin 1 (TIA‐1) molecules and increased NK cytotoxicity against resistant Daudi cells.^53^ Thus, while mediating tolerance and escape from adaptive immunity, IL‐10 cytokine may promote NK cells' antitumor activity in early stages. Thus, the rapid progression and high levels of IL‐10 secreted by group 2 and group 3 patients after BTZ + TMZ treatment may have acutely induced their activated NK cell phenotype.

However, such a substantial Th2 immune response is likely to down‐regulate tumor‐specific Th1 and CD8^+^T cell responses^54^ in the long run and contribute to tumor immune evasion and cancer progression in these patients. CTLA‐4 expression would further decrease T cell activation, proliferation and effector functions.^55^, ^56^ In contrast, group 1 patients with lower disease burden and slower tumor growth kinetics had less suppressive Th2 driven cytokine responses and specifically IL‐10, and expanded CD4^+^ effector T cells in response to treatment. We speculate that this reflects beneficial Th1 and CD8^+^ T cell mediated antitumor responses enhanced by BTZ + TMZ combination therapy, possibly due to enhanced tumor antigen presentation.

Surprisingly, group 3 patients with mixed clinical/radiological responses had the lowest tumor burden (ca. 5.5 × 10^3^ mm^3^) yet exhibited the most rapid population doubling time of 35.1 days. These patients also had multiple neoplastic lesions. In response to BTZ + TMZ treatment, group 3 patients secreted the highest concentrations of immunosuppressive IL‐10 cytokine in plasma and exhibited a tolerogenic phenotype (partially overlapping with the group 2 patients) denoted by upregulated PD‐1^+^CD4^+^ and CTLA‐4 ^+^CD8^+^ T cell subsets compared to group 1 patients. TMZ can suppress immune responses in some glioma patients as it has been shown to downregulate activation of the JAK/STAT pathway and induce PD‐L1 expression on tumor cells.^57^

Although group 2 and 3 patients responded to PMA/ionomycin stimulation by secreting high levels of the IFNγ Th1 cytokine, their levels of IL‐10 were many magnitudes higher, culminating in an overall immune suppressive phenotype. These data might indicate that the tolerant phenotype is not simply a feature of high tumor burden but may reflect tumor cell intrinsic features as the tumor leverages multipronged strategies to escape immune detection and destruction.^58^ The caveat to this study is the small sample size and lack of randomized controls as it was powered primarily for evaluation of safety and tolerance of sequentially combined BTZ with dose escalated TMZ, as well as radiological and immunological biomarker analyses. The latter was challenged by the unavailability of sufficient ex vivo PBMCs for NK cell isolation and fresh autologous tumor cells from all patients for functional assessment.

Nevertheless, cytokine signaling locally in the brain cross‐talks with that in the systemic circulation, and aberrant function is often reflected in unison with phenotypic defects. In conclusion, 3 of 4 patients in group 2 were recruited into the BORTEM‐17 trial after their first tumor recurrence and this distinguished them from both group 1 and group 3 patients where all patients were recruited after their second recruitment. Group 1 patients further distinguish from both group 2 and group3 by diminished IL‐10 levels. Thus, levels of IL‐10 after BTZ + TMZ treatment could be monitored prospectively as a potential biomarker for probability of immunological responses defining the patients as group 1‐, 2‐, or 3‐type. Combination BTZ + TMZ treatment was safe, tolerated and immunological and MRI biomarkers provided robust indication for best responder patients exhibiting Th1 driven immune responses.

## CONFLICT OF INTERESTS

The authors declare that there are no conflict of interests.

## AUTHOR CONTRIBUTIONS

Conducted experiments/analysed data: MAR, JB, AGN, VA, MH, AW, HM, JH, LO, LH, SAL, and AL. Treated and followed‐up patients: JB, DG, and PB. Writing the manuscript: MAR, VA, and MC. Revised manuscript: all authors. Designed research: MC and DG. Acquired funding: MC

## Supporting information


**Supplementary Figure 1**. *Fluorescence‐minus‐one (FMO) controls for the selected antibodies form the panel of antibody used in this study*. Dot plots of multicolor flow cytometry showing the fluorescence spread into the channels shown by the FMO control compared to an unstained control, right most graphs show fully stained PBMC samples. The graphs show the FMO for (A) BV570 CD4, (B) PE Cy7 CD8, (C) PE PD‐1, (D) BV786 CTLA4, (E) APC CD45RO and (F) V450 CD56. Blue line represents FMO gating boundary

**Supplementary Figure 2**. *Bortezomib induces NK cell maturation phenotypes and expression of inhibitory checkpoints*. Representative dot plots showing CD56 *vs*. CD16 expression within the CD56^+^ CD3^‐^ NK cell gate. (**B**) Mean ± S.E.M. % of CD56 bright NK cell subsets in group 1, 2 and 3 patients before, during and after treatment with BTZ and TMZ. (**C**) Representative dot plots showing CD57 expression within the CD56 gate. (**D**) Mean ± S.E.M. % of CD16^+^ NK cell subsets in group 1, 2 and 3 patients before, during and after treatment with BTZ and TMZ. Mean ± S.E.M % of (**E**) CD57, (**F**) NKG2A, (**G**) PD‐1, (**H**) DNAM‐1, and (**I**) CD69 expression in NK cells from group 1, 2 and 3 patients, before, during and after treatment with BTZ and TMZ. Data represents the mean ± S.E.M. of n = 10 patients. Two‐way ANOVA, Bonferroni's multiple comparison test, * *P* < 0.05; ** *P* < 0.01; *** *P* < 0.001: **** *P* < 0.0001

Supporting information

Supporting information
