# Supplementary figures and images for: Sequential bortezomib and temozolomide treatment promotes immunological responses in glioblastoma patients with positive clinical outcomes: A phase 1B study
Source: Immun Inflamm Dis. 2020 Jun 24;8(3):342–59. doi: 10.1002/iid3.315 (PMC7416034; doi:10.1002/iid3.315)

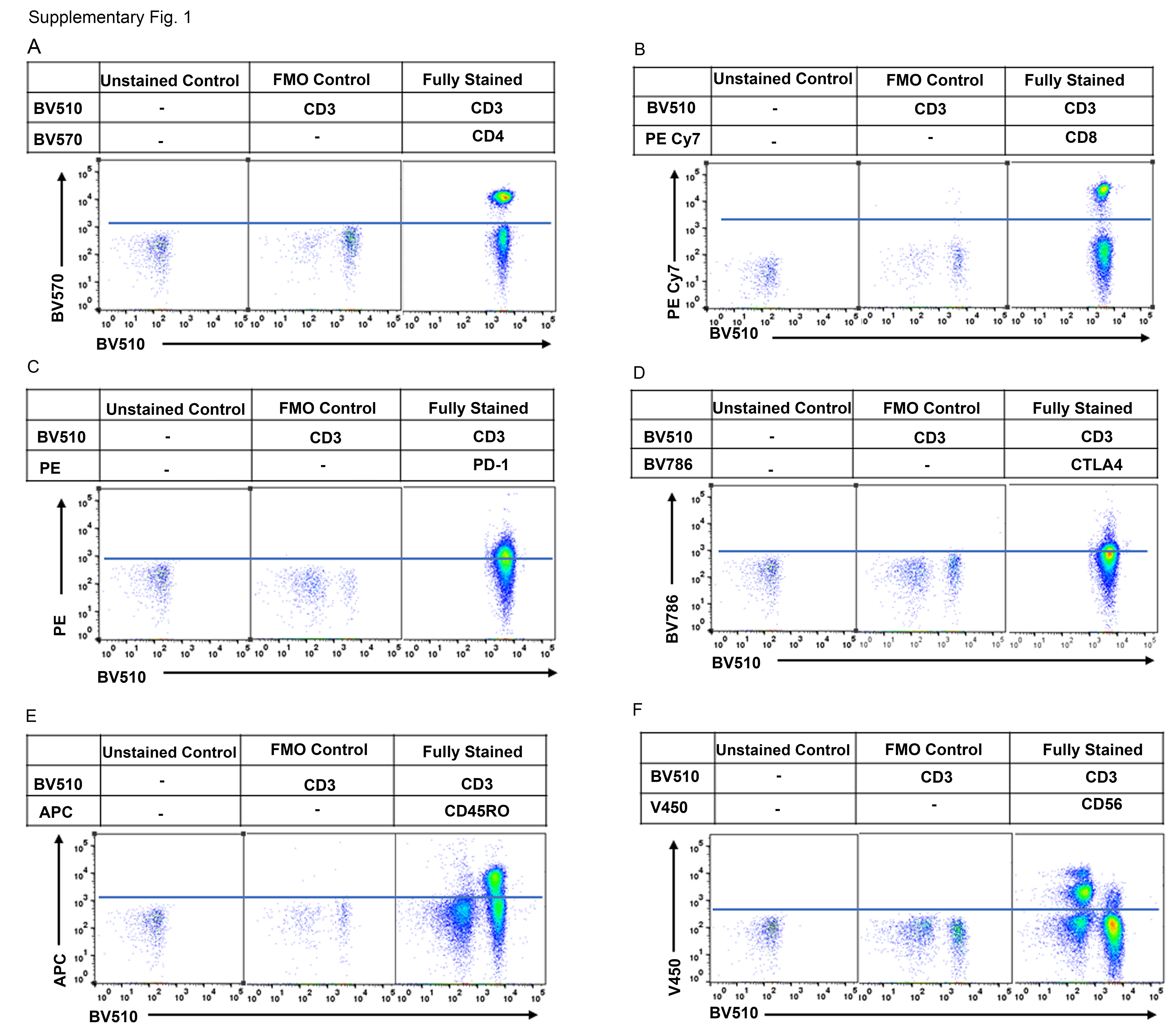

Supplement: Supplementary file 1 — Supplementary Figure 1. Fluorescence‐minus‐one (FMO) controls for the selected antibodies form the panel of antibody used in this study. Dot plots of multicolor flow cytometry showing the fluorescence spread into the channels shown by the FMO control compared to an unstained control, right most graphs show fully stained PBMC samples. The graphs show the FMO for (A) BV570 CD4, (B) PE Cy7 CD8, (C) PE PD‐1, (D) BV786 CTLA4, (E) APC CD45RO and (F) V450 CD56. Blue line represents FMO gating boundary [file IID3-8-342-s001.tif]

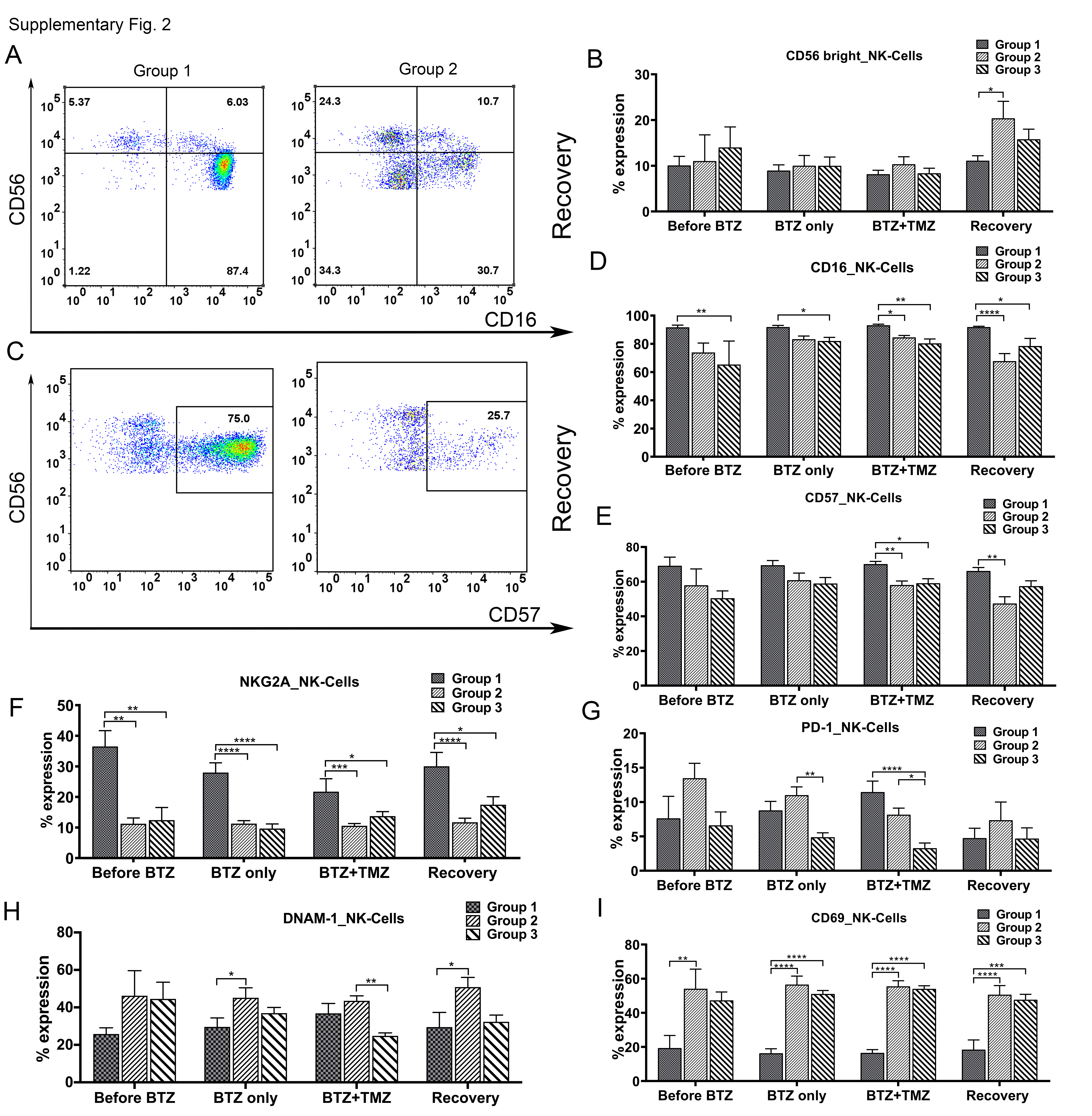

Supplement: Supplementary file 2 — Supplementary Figure 2. Bortezomib induces NK cell maturation phenotypes and expression of inhibitory checkpoints. Representative dot plots showing CD56 vs. CD16 expression within the CD56+ CD3‐ NK cell gate. (B) Mean ± S.E.M. % of CD56 bright NK cell subsets in group 1, 2 and 3 patients before, during and after treatment with BTZ and TMZ. (C) Representative dot plots showing CD57 expression within the CD56 gate. (D) Mean ± S.E.M. % of CD16+ NK cell subsets in group 1, 2 and 3 patients before, during and after treatment with BTZ and TMZ. Mean ± S.E.M % of (E) CD57, (F) NKG2A, (G) PD‐1, (H) DNAM‐1, and (I) CD69 expression in NK cells from group 1, 2 and 3 patients, before, during and after treatment with BTZ and TMZ. Data represents the mean ± S.E.M. of n = 10 patients. Two‐way ANOVA, Bonferroni's multiple comparison test, * P < 0.05; ** P < 0.01; *** P < 0.001: **** P < 0.0001 [file IID3-8-342-s002.tif]
