# Supplementary material for: Sequential bortezomib and temozolomide treatment promotes immunological responses in glioblastoma patients with positive clinical outcomes: A phase 1B study
Source: Immun Inflamm Dis. 2020 Jun 24;8(3):342–59. doi: 10.1002/iid3.315 (PMC7416034; doi:10.1002/iid3.315)
Supplement: Supplementary file 3 — Supporting information [file IID3-8-342-s003.doc]

Table S1: List of antibodies used in flow cytometry phenotyping

| **Ab** | **Fluorochrome** | **Catalog number** |  | **Anti-** | **Isotype** | **Clone** | **Brand** |
| --- | --- | --- | --- | --- | --- | --- | --- |
| **CD56 (NCAM)** | V450 | 560360 | Mouse | Human | IgG1, ƙ | B159 | BD Bioscience (Trondheim, Norway) |
| **CD16** | FITC | 555406 | Mouse | Human | IgG1, ƙ | 3G8 | BD Bioscience (Trondheim, Norway) |
| **NKG2A (CD159a)** | Alexa700 | FAB1059N-100 | Mouse | Human | IgG2a | 131411 | R&D systems (Abingdon, UK) |
| **DNAM-1 (CD226)** | PerCP/Cy5.5 | 338314 | Mouse | Human | IgG1, ƙ | 11A8 | BioLegend, (Oslo, Norway) |
| **PD-1 (CD279)** | APC | 558694 | Mouse | Human | IgG1, ƙ | MIH4 | BD Bioscience (Trondheim, Norway) |
| **PD-1 (CD279)** | PE | 557946 | Mouse | Human | IgG1, ƙ | MIH4 | BD Bioscience (Trondheim, Norway) |
| **CD69** | PE | 555531 | Mouse | Human | IgG1, ƙ | FN50 | BD Bioscience (Trondheim, Norway) |
| **CD57** | PE-Cy5 | 9665-13 | Mouse | Human | IgM | NK-1 | SouthernBiotech ( Birmingham, AL, USA) |
| **CD3** | V500 | 560770 | Mouse | Human | IgG1, ƙ | SP34-2 | BD Bioscience (Trondheim, Norway) |
| **CD4** | BV570 | 300534 | Mouse | Human | IgG1, ƙ | RPA-T4 | BioLegend, (Oslo, Norway) |
| **CD8** | PECy7 | 557746 | Mouse | Human | IgG1, ƙ | RPA-T8 | BD Bioscience (Trondheim, Norway) |
| **CCR7 (CD197)** | BV711 | 353228 | Mouse | Human | IgG2a, ƙ | G043H7 | BioLegend, (Oslo, Norway) |
| **CD45RO** | APC | 130-095-460 | Mouse | Human | IgG2a, ƙ | UCHL1 | Miltenyi (Gladbach, Germany) |
| **CTLA-4 (CD152)** | BV786 | 563931 | Mouse | Human | IgG2a, ƙ | BNI3 | BD Bioscience (Trondheim, Norway) |
| **LiveDead** | Near-IR (APCCy7) | L34976 | - | - | - | - | Invitrogen (Hämeenlinna, Finland) |
