# Supplementary material for: Sequential bortezomib and temozolomide treatment promotes immunological responses in glioblastoma patients with positive clinical outcomes: A phase 1B study
Source: Immun Inflamm Dis. 2020 Jun 24;8(3):342–59. doi: 10.1002/iid3.315 (PMC7416034; doi:10.1002/iid3.315)
Supplement: Supplementary file 4 — Supporting information [file IID3-8-342-s004.docx]

*Patient eligibility*

The study protocol (Fig. 1A) was approved by the regional ethical board for Western Norway (2017/2084) and the Norwegian Medicines Agency (17/17445-17). All eligible patients signed the approved consent form for study participation before undergoing any study related procedures. Eligible patients were 18 years or older with histological and molecular pathology confirmed GBM with unmethylated *MGMT* promoter and a life expectancy greater than 8 weeks. They should have Karnofsky performance status ≥ 70 and no peripheral neuropathy ≥ grade 2. Haematological criteria for inclusion were white blood cell count (WBC) ≥ 3,000/mm^3^; absolute neutrophil count (ANC) ≥ 1,500/mm^3^; platelet count ≥ 100,000/mm^3^; haemoglobin ≥ 10 g/dL, bilirubin < 2.5 times upper limit of normal (ULN); serum aspartate aminotransferase (ASAT) < 2.5 times ULN; estimated glomerular filtration rate (GFR) ≥ 60 mL/minute, serum sodium level > 130 mmol/L and serum potassium level within normal limits. Eligible patients had magnetic resonance imaging (MRI) confirmed tumour relapse or progression ≥ 12 weeks after completed radiotherapy evaluated based on criteria defined by Response Assessment in Neuro-oncology (RANO) [1]. Additionally, the tumour could not be available for stereotactic radiosurgery (SRS). However, patients that were previously treated with SRS were eligible for inclusion, providing the presence of a measurable neoplastic contrast-enhancing lesion outside the irradiated area. An unstained paraffin block or cryopreserved tumour tissue from surgical resection was required for quantitative *MGMT* methylation status by sodium bisulphite conversion and pyrosequencing. Previous treatment with bevacizumab or lomustine, procarbazine and vincristine (PCV) for relapsed glioblastoma was not permitted. Patients on CYP450 or -3A4 enzyme inducing anti-epileptic drugs (EIAED) had to be transitioned to non-EAIED for ≥ 2 weeks prior to enrolment since bortezomib is a substrate of several isoenzymes in the cytochrome P450 system[2,3]. Unfractionated and/or low molecular weight heparin was permitted but stable or decreasing steroid dose two weeks before study entry was a requirement. Patients with hypersensitivity to bortezomib, boron, or mannitol and/or contraindications for temozolomide were excluded. A negative pregnancy test no later than 14 days prior to enrolment was required for fertile female patients. Likewise, their male partners of childbearing potential were required to use adequate contraception. Patients were excluded if they had a history of New York Heart Association (NYHA) class III or IV heart failure, uncontrolled angina, severe electrocardiographic ventricular arrhythmias, myocardial infarction/ischemia or known heart failure within the past 6 months. Patients were also excluded if they had a history of serious medical or psychiatric illness that could interfere with study consent and compliance. These conditions included, but were not limited to, active infection requiring intravenous antibiotics, immunocompromised states (e.g., human immuno-deficient virus, systemic lupus erythematosus), active viral hepatitis (HBV surface antigen positive) or hepatitis C infection, stroke within the past 6 months or other malignancy within the past 3 years. Psychiatric illness and/or social situations that at the doctor’s discretion could limit compliance with study requirements, or disease that could obscure toxicity or dangerously alter drug metabolism were also causes for exclusion. All other investigational drugs had to be stopped at least 12 weeks prior to enrolment. The study was registered with the clinicaltrial.gov identifier (NCT03643549).

*Screened but not enrolled patients*

A total of 16 patients with suspected recurrent GBM were screened for enrolment into the phase IB study from August 2018 to October 2019 at Haukeland University Hospital, Norway. Six patients were screened but not enrolled due to rapid clinical deterioration (2 male patients aged 54yrs and 64yrs), poor performance status (male patient, age 66yrs), previous PCV therapy for relapsed GBM (male patient 18yrs), personal reasons (female, 64yrs) and no radiologically confirmed tumour progression despite clinical deterioration (female 58yrs).

*MRI sequences and Quantification of radiologic tumour growth*

The MRI scanning protocol included sagittal 3D T1-weighted MPRAGE pre-contrast (isotropic 1x1x1 mm^3^ voxels), axial T2-weighted TSE (0.43x0.43x4.80 mm^3^ voxels), oblique T2-weighted FLAIR (0.49x0.49x0.90 mm^3^ voxels) and a sagittal 3D T1-weighted MPRAGE post-contrast series, the latter was used for volumetric analysis. Contrast agent dose was calculated based on patients’ body weight. The entire scanning protocol, also including a T2* perfusion series, multiple flip-angle 3D FLASH recordings for T1-mapping, and a RESOLVE diffusion-weighted recording for ADC mapping (not reported here), was completed within 1hr.

To visualize tumour progression *in situ*, we applied rigid multiresolution co-registration between the 3D T1-weighted post-contrast acquisition at time t=t0 (fixed image) and the successive recordings at times t=ti (moving image, where i=1,2,3) to correct for translation and rotational movement of the head between the MRI sessions using functionality from the Elastix toolbox (<http://elastix.isi.uu.nl/>). The volumetric boundaries of the tumour were defined using manual pseudolabeling in ITK-SNAP version 3.6.0 [4] by in-painting the regions of interest (ROIs) slice-by-slice on axial slices. The delineation included all contrast enhanced areas of the tumour, excluding the surgical resection cavity but included any residual enhancing tumour localized along the resection cavity boundary. The window level (WL) and window width (WW) of all images was kept constant and unchanged throughout the course of the delineation process. Linear mixed effects regression model was used to analyse the longitudinal tumour volumes. Time (slope) was included as a continuous variable, group was a categorical variable and patients were classified as random effects using Stata software, version 15.1 (College Station, Texas, USA). Tumour volume doubling time was calculated using the following equation:

$$Doubling time (days) =\frac{duration (days) * log(2)}{\log\left( tumor volume day 56 \right)/\log(baseline tumor volume)}$$

*Bortezomib pharmacokinetics*

Patients’ blood was collected in a time course during the first treatment cycle, before bortezomib administration at t=0h, and thereafter at 0.5h, 1h, 4h, 8h and 24h post bortezomib bolus injection on day 1 (bortezomib only) and at t=0h, and thereafter at 0.5h, 1h, 4h and 8h on day 7 (bortezomib + TMZ). The blood drawn into EDTA and sodium heparin containing tubes was immediately centrifuged at 2000xg for 10min at 4°C to obtain plasma. After plasma extraction, the blood was diluted in phosphate buffered saline and leucocytes obtained by further centrifugation in lymphocyte solution in SepMate50 tubes (Corning, NY, USA) following standard procedures[5]. Plasma and enumerated leucocytes were aliquoted into vials for cryopreservation or Eppendorf tubes for further analyses. For bortezomib pharmacokinetic analysis, 10μl of formic acid (Merck) was added to 500μl of plasma obtained from the sodium heparin tubes on ice and within 30min after blood draw in accordance with the protocol provided by Covance Inc. (Oxford, UK). The concentration of bortezomib in plasma was quantified by liquid chromatography and tandem mass spectrometry. To estimate C_p_ at t=0, we performed exponential regression analyses based on the following equation:

$$C_{pt}=C_{max}\cdot e^{-kt}$$

Where C_pt_ is plasma drug concentration at a given time (t), C_max_ is plasma concentration at t=0, *k* is distribution or elimination rate constant. To calculate AUC, C_max_ was estimated by extrapolation to t=0, based on time-points 0.5 and 1 h. Since no BTZ was detected in the 24h samples, these were omitted from the calculations.

*GBM tissue dissociation*

Fresh biopsy tissue from primary operation of patient 02was mechanically diced with scalpels and further dissociated into single-cell suspensions in 5% Liberase DH (Roche Molecular Diagnostics; Pleasanton, CA, USA) and 2% deoxyribonuclease I (Worthington) in HBSS (Gibco, Thermo Fisher Scientific; Waltham, MA, USA) at 37°C for 30 min in a shaking water bath at 220 strokes/min, through successive rounds. Cells were triturated, filtered through a 100µm cell strainer (BD Falcon), and centrifuged at 900 rpm for 5 min for harvesting. Cell filtering was repeated once over a 70 µm cell strainer (BD Falcon) followed by twice over 40 µm filters (BD Falcon).

*DNA extraction from FFPE tissues and pyrosequencing*

Formalin fixed paraffin embedded (FFPE) tumour tissue blocks were sectioned into 3 μm thick sections and stained with hematoxylin and eosin to determine tumour cellularity. Sections with at least 80% tumour cells were selected for DNA extraction. DNA was isolated using the DNA FFPE tissue kit (Qiagen, Crawley, UK) following the manufacturer's instructions. *MGMT* promoter methylation was quantified using pyrosequencing as previously described [6].

*Flow cytometry and Ex vivo stimulation and cytokine analysis*

PBMCs purified from buffy coats were counted, diluted to 0.5 x10^6^ cells/1ml FACs buffer per tube and stained with fluorescent antibodies. Staining specificity was confirmed using Fluorescent Minus One (FMO) controls for each channel. Samples were incubated in the dark for 30 min at 4º and thereafter washed with FACs buffer by centrifugation at 300xg for 5min at 4°C. For tubes requiring secondary antibodies, 100µl of the antibody stocks were added to the corresponding tubes and further incubated in the dark for 20min at 4°C. At least 300.000 events/tube were acquired on BD LSR FORTESSA (BD Biosciences, Trondheim, Norway) and analysed with FlowJo software version 10 (Tree Star Inc.; Ashland, OR, USA). For the gating strategy, debris and doublets were excluded using SSC and FSC, then singlets and live cells gated on the FSC *vs*. Live/Dead and then dot plots on the FSC-H *vs*. FSC-W were selected for further analysis. *Ex vivo stimulation and cytokine analysis* is detailed in Supplementary information.

To assess cytokine production, 0.5 x10^6^ PBMCs were thawed and resuspended in 500µl RPMI culture medium without serum. The cells were stimulated with phorbol 12-myristate 13-acetate (PMA 20 ng/ml) (Sigma-Aldrich, Oakville, ON, Canada) and ionomycin (500 ng/ml) (Sigma-Aldrich) or left unstimulated as negative control, in the presence of brefeldin A (5 ug/ml) (Sigma-Aldrich) for 5hrs before flow cytometry analysis as previously described[7]. To assess degranulation, PBMCs were stimulated with autologous tumour cells (P02) or PMA/ionomycin in the presence of monensin (1 mM) (Sigma-Aldrich) and CD107a antibody (BD Biosciences) or corresponding isotype for 5hrs at 37°C in 5% CO_2._ Thereafter cells were harvested and stained with fluorescent conjugated antibodies recognising surface markers (Table S2), prior to fixation in Cytoperm/Cytofix and staining for intracellular markers. Data was acquired on BD LSR FORTESSA (BD Biosciences) and analysed with FlowJo software version 10, (Tree Star Inc.) Cytokines present in patients’ plasma aliquots from various treatment time points, as well as pooled supernatants from each independent *ex vivo* stimulation experiment were analysed with Luminex-based enzyme linked immunosorbent assay (ELISA) with a kit that detected 17 analytes (Merck Millipore, Darmstadt, Germany) according to the manufacturer’s instructions and as previously described[8].

References

1. Wen, P.Y.; Macdonald, D.R.; Reardon, D.A.; Cloughesy, T.F.; Sorensen, A.G.; Galanis, E.; Degroot, J.; Wick, W.; Gilbert, M.R.; Lassman, A.B., et al. Updated response assessment criteria for high-grade gliomas: response assessment in neuro-oncology working group. *J Clin Oncol* **2010**, *28*, 1963-1972, doi:10.1200/JCO.2009.26.3541.

2. Pekol, T.; Daniels, J.S.; Labutti, J.; Parsons, I.; Nix, D.; Baronas, E.; Hsieh, F.; Gan, L.S.; Miwa, G. Human metabolism of the proteasome inhibitor bortezomib: identification of circulating metabolites. *Drug Metab Dispos* **2005**, *33*, 771-777, doi:10.1124/dmd.104.002956.

3. Uttamsingh, V.; Lu, C.; Miwa, G.; Gan, L.S. Relative contributions of the five major human cytochromes P450, 1A2, 2C9, 2C19, 2D6, and 3A4, to the hepatic metabolism of the proteasome inhibitor bortezomib. *Drug Metab Dispos* **2005**, *33*, 1723-1728, doi:10.1124/dmd.105.005710.

4. Yushkevich, P.A.; Piven, J.; Hazlett, H.C.; Smith, R.G.; Ho, S.; Gee, J.C.; Gerig, G. User-guided 3D active contour segmentation of anatomical structures: significantly improved efficiency and reliability. *Neuroimage* **2006**, *31*, 1116-1128, doi:10.1016/j.neuroimage.2006.01.015.

5. Dominguez-Valentin, M.; Gras Navarro, A.; Rahman, A.M.; Kumar, S.; Retiere, C.; Ulvestad, E.; Kristensen, V.; Lund-Johansen, M.; Lie, B.A.; Enger, P.O., et al. Identification of a Natural Killer Cell Receptor Allele That Prolongs Survival of Cytomegalovirus-Positive Glioblastoma Patients. *Cancer Res* **2016**, *76*, 5326-5336, doi:10.1158/0008-5472.CAN-16-1162.

6. Mikeska, T.; Bock, C.; El-Maarri, O.; Hubner, A.; Ehrentraut, D.; Schramm, J.; Felsberg, J.; Kahl, P.; Buttner, R.; Pietsch, T., et al. Optimization of quantitative MGMT promoter methylation analysis using pyrosequencing and combined bisulfite restriction analysis. *J Mol Diagn* **2007**, *9*, 368-381, doi:10.2353/jmoldx.2007.060167.

7. Clenet, M.L.; Gagnon, F.; Moratalla, A.C.; Viel, E.C.; Arbour, N. Peripheral human CD4(+)CD8(+) T lymphocytes exhibit a memory phenotype and enhanced responses to IL-2, IL-7 and IL-15. *Sci Rep* **2017**, *7*, 11612, doi:10.1038/s41598-017-11926-2.

8. Gras Navarro, A.; Kmiecik, J.; Leiss, L.; Zelkowski, M.; Engelsen, A.; Bruserud, O.; Zimmer, J.; Enger, P.O.; Chekenya, M. NK cells with KIR2DS2 immunogenotype have a functional activation advantage to efficiently kill glioblastoma and prolong animal survival. *J Immunol* **2014**, *193*, 6192-6206, doi:10.4049/jimmunol.1400859.
